# Pyrethroid-Induced Organ Toxicity and Anti-Oxidant-Supplemented Amelioration of Toxicity and Organ Damage: The Protective Roles of Ascorbic Acid and α-Tocopherol

**DOI:** 10.3390/ijerph17176177

**Published:** 2020-08-25

**Authors:** Mohsen S. Al-Omar, Mamuna Naz, Salman A. A. Mohammed, Momina Mansha, Mohd N. Ansari, Najeeb U. Rehman, Mehnaz Kamal, Hamdoon A. Mohammed, Mohammad Yusuf, Abubaker M. Hamad, Naseem Akhtar, Riaz A. Khan

**Affiliations:** 1 Department of Medicinal Chemistry and Pharmacognosy, College of Pharmacy, Qassim University, Qassim 51452, Saudi Arabia; m.omar@qu.edu.sa (M.S.A.-O.); ham.mohammed@qu.edu.sa (H.A.M.); 2 Department of Medicinal Chemistry and Pharmacognosy, Faculty of Pharmacy, Jordan University of Science and Technology, Irbid 22110, Jordan; 3 Department of Pharmacology and Toxicology, College of Pharmacy, Prince Sattam Bin Abdulaziz University, Al-Kharj 11942, Saudi Arabia; mona.mansha@hotmail.com (M.N.); nazam.ansari@gmail.com (M.N.A.); n_rehman5@hotmail.com (N.U.R.); 4 Department of Pharmacology and Toxicology, College of Pharmacy, Qassim University, Qassim 51452, Saudi Arabia; 5 Department of Microbiology, Faculty of Biological Sciences, Quaid-e-Azam University, Islamabad 15320, Pakistan; momina.mansha@hotmail.com; 6 Department of Pharmaceutical Chemistry, College of Pharmacy, Prince Sattam Bin Abdulaziz University, Al-Kharj 11942, Saudi Arabia; mailtomehnaz@gmail.com; 7 Department of Pharmacognosy, Faculty of Pharmacy, Al Azhar University, Cairo 11371, Egypt; 8 Department of Clinical Pharmacy, College of Pharmacy, Taif University, Taif 21974, Saudi Arabia; yusuf4682@gmail.com; 9 Department of Basic Sciences, Preparatory Year Program, Prince Sattam Bin Abdulaziz University, Al-Kharj 11942, Saudi Arabia; abkr.hamad@gmail.com; 10 College of Dentistry and Pharmacy, Buraydah Private Colleges, Buraydah, Qassim 51488, Saudi Arabia; nzansar@gmail.com

**Keywords:** pyrethroids, ascorbic acid, α-tocopherol, organ damage, histopathology, toxicity amelioration, antioxidants

## Abstract

The pyrethroid toxicants, fatal at high doses, are found as remnants of crop pesticides and ingredients of commercially available insecticides. The toxic effects of high-content insecticidal pyrethroid formulations are available in 0.05 g, 1.17 g, and 0.04 g pyrethroid-instilled products, namely burning coils, pyrethroid-soaked mats, and liquid formulations of pyrethroids that release pyrethroid vapor/smoke upon heating. They provided 5.46 g/kg, 21.15 g/kg, and 4.24 g/kg of toxicants to the experimental animals over a total of 3 weeks/5 h per os (p.o.) administration, producing necrosis, hyperemia, and fatty changes in the liver; fiber separation in cardiac muscles; atrophy, lymphatic infiltration, blood vessel congestion, and hyperemia in the heart tissues of the experimental animals. The glomerular tuft necrosis, cytoplasmic degeneration of renal tubular cells, necrotic tubules, congestion, and dilatation of blood vessels were observed in the kidney tissue of intoxicated animals. Air-space enlargement, interstitial inflammation, lymphocyte infiltration aggregates, connective tissue infiltration by inflammatory cells, and hyperemia were found in the lung tissues. The pyrethroid toxicants also produced nervous tissue degeneration and decreased neurons in the brain, which were observed through histopathological examinations of the brain, lungs, heart, kidneys, and liver. The protective effects of ascorbic acid (AA/vitamin C) and α-tocopherol (E307/vitamin E) at 100 mg/kg oral doses administered daily for the entire period of the toxicant exposure of three weeks to the experimental mice, aged between 3–4 months and weighing ≈30 g, ameliorated the tissue damage, as observed through the histopathological examinations. The ascorbic acid caused recovery of the liver, kidney, brain, and heart tissue damage, while α-tocopherol was effective at ameliorating the damage in the kidneys and lung tissue compared with the control groups. The high levels of tissue damage recovery suggested a prophylactic effect of the concurrent use of ascorbic acid and α-tocopherol for the subjects under the exposure of pyrethroids.

## 1. Introduction

Pyrethroid toxicants are found in contaminated fruits, vegetables, and other foodstuffs, including grains as remnants of crops, produce after pyrethroid pesticide use [1], and as ingredients of various commercially available insecticidal products [2]. Excessive pyrethroid ingredients are still encountered in crops from certain farming communities, although crop-processing helps to decrease the levels of pyrethroid contamination through evaporation, washing, and sunlight exposure. Nonetheless, the environmental presence and remnants of pyrethroid pesticide levels above allowed safe limits are still encountered, especially in developing countries of Asia and Africa, where natural, and, more prevalently, synthetic pyrethroids have been abundantly used for crop protection for years [3]. The practice raises the pyrethroids’ eventual concentrations in the soil, environment, exposed entities, foodstuffs, and their storage areas, together with exposures to humans. Unexpectedly high and alarming levels of pyrethroid residues have recently been detected in the environment, including in sediment samples and agricultural land areas from developed countries [4]. In this context, the presence of pesticidal residues in the environment, especially regarding exposures in the residential and other habitable localities, produce infiltration-induced side effects in humans, exposed children, pregnant women, and farm and garden/orchard workers need more attention [5]. Another source of contamination of pyrethroids on a large scale is humans’ use of pesticidal ingredients, such as mosquito repellants in underdeveloped and developing countries [6]. The commercially available pyrethroid products release vaporizable toxicants upon vaporization through slight heating or slow-burning that may produce severe tissue damage above a specified level of vapor intake, as in the case of continued overnight exposures from the mosquito repellant products. The continued exposure may also affect vital organs, leading to organ malfunction and cause high toxicity in the affected subjects at temporary and permanent timescales depending upon the duration and dose of the toxicants [7]. These pyrethroids’ ingredients also interfere with the body’s natural hormone functioning, homeostasis, and above all, immunity. These ingredients are also suspected to decrease fertility, increase cancer risks, and make changes in a severely exposed adult person’s behavior [8,9].

**Figure 1:**
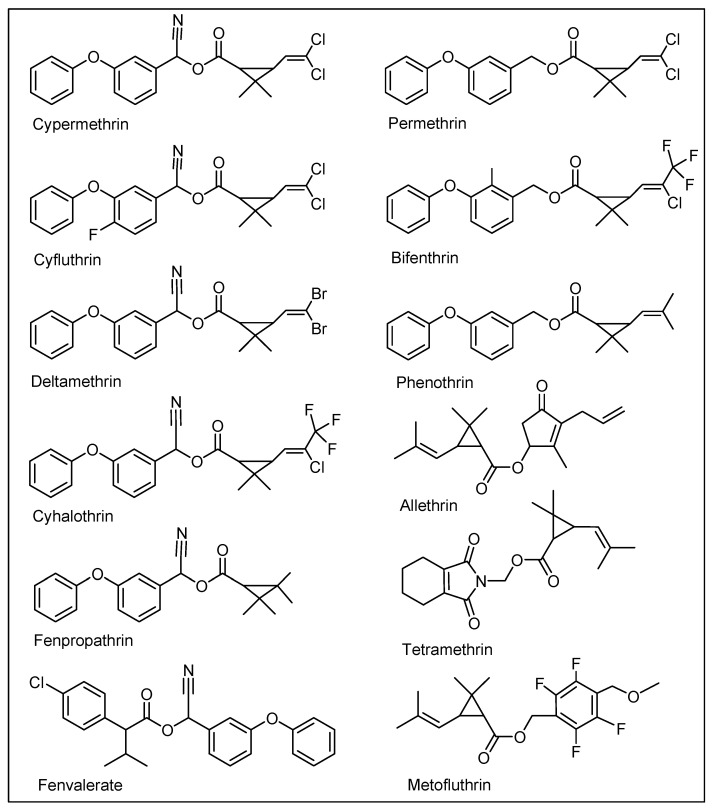
Chemical structures of major cyano, and non-cyano pyrethrins.

The risk factors involved are severe and the prevention and amelioration of the toxic effects of the pyrethroid ingredients for the affected humans and other subjects, including sanitization of their habitat and clean-up of their overall environment, are required. The pyrethroids’ pesticidal formulations usually contain active ingredient at 0.3–0.5% *w*/*v* pyrethroids per liter, which on the industrial farming scale is quite high and risky for humans, animals, and the environment. The knockdown doses for pests and insects are low but the scale of usage puts the amounts at alarmingly higher levels, and without doubt, is certainly a cause for concern. The commercial products of insect repellants, i.e., coil, liquids, and mat-absorbed pyrethroid products, contain 0.05 g/coil, 2.6 g/100 mL, and 0.04 g/mat as the effective pyrethroid concentrations, respectively, which during continued use to repel mosquitoes, especially during the summer season, amounts to higher degrees of exposure. In particular, the liquid formulation levels of exposure are nearer to the exposure of farmworkers who are using a pyrethroid spray. However, the rapid metabolism of pyrethroids in humans has restricted the eventual and immediate onset of toxicity, but exposure at broader, higher, and continuous scales, and during intensive seasonal usage, is a cause for concern. The metabolic and environmental degradations of pyrethroids are structure-sensitive and favored as the first generation of synthetic pyrethroid-derived/based pyrethrins structures, i.e., the natural pyrethroid products sourced from pyrethrums (*Chrysanthemum cinerariaefolium* and *Chrysanthemum coccineum),* which are not commonly in use today as either agriculture pesticides or as a household insecticide. The second generation of pyrethroids are more stable, degradation-resistant, technical grade (concentrated), synthetic pyrethroids, which are in frequent use and are comparatively more toxic than the natural pyrethroids to humans, other vertebrates, and invertebrates, including the insects with beneficial roles, e.g., bees, dragonflies, and mayflies. The oral exposure limits for humans for various pyrethroids ranges between 0.005–0.05 mg/kg/day, depending upon the type of pyrethroid products. However, in the United States, the average exposure limit for a young male adult (70 kg) is set at 3.2 µg/per day for permethrin, an abundantly used pyrethroid in the US [10], which is very low compared to the World Health Organization limit for drinking water at 20 µg/L. The occupational exposure limit, according to OSHA (Occupational Safety and Health Administration, USA), is set at 5 mg/m^3^/8 h/day/40 h weekly [11]. The toxicity is a manifestation of the dose, duration, and mode of exposure, personal habits, and presence of non-pesticidal synergist products in the pyrethrin formulation. Structurally, the synthetic pyrethroid analogs of second-generation developments are categorized as either type I or II, where Type I contains an ester linkage without a cyano (C≡N) group; this type of products includes allethrin, permethrin, phenothrin, bifenthrin, metofluthrin, and tetramethrin. In contrast, the Type II products contain a cyano group and an ester linkage. It includes deltamethrin, fenvalerate, cyfluthrin, cyhalothrin, fenpropathrin, and cypermethrin. The presence of a cyclopropyl ring can be common to both types of structures [12] (Figure 1). These pyrethroids are stereo-chemically defined products. However, the active principle(s) in pesticidal liquid and insecticide formulations can also be a mixture of natural pyrethrins directly extracted from the plant sources, or allethrin, metfluthrin, and cyphenothrin pyrethroids, the later developed synthetic analogs. These pyrethroids also severely harm insects and aquatic life at lower to moderate doses, and they target humans and other mammalian species at moderate to higher doses depending upon their structural susceptibility and systemic metabolism, where studied exposure/inhalation limits have been set. The pyrethroid products, alone and in combination with other toxicity-enhancing synergist molecules, e.g., piperonyl butoxide and *N*-octyl bicycloheptene dicarboximide (MGK-264), can be severely toxic and fatal at higher doses [13,14]. However, the biosystem’s remediation capacity and detoxifying bio-actions at lower concentrations of these toxicants limit the toxicity manifestations in humans and vertebrate animals [15,16]. Given the fact that the pyrethroids’ active components are responsible for severe adverse effects and toxicological manifestations in individuals [17], the exposure via intake or inhalation of the vapor of these toxic ingredients causes morphological changes in the alveolar cells and the metaplasia of the epithelial cells, which are among some of the most commonly reported undesirable systemic effects. The pyrethroid constituents cause multifocal congestion, interstitial mononuclear cellular infiltration, cystic dilation in the medulla, and extensive fibrosis in affected animals’ kidneys. These toxicants are also known to cause hyperemia, degeneration, and atrophy in cardiac tissues, with extensive noxious effects on the nervous system [18,19,20,21,22].

The current study intended to explore the histopathological changes and their remediation in different organs of the experimental animals, i.e., mice exposed to pyrethroids, which humans may encounter when exposed in agro-sector activities, as well as being inhaled from uses of commercially available pyrethroid-formulated products, such as household insecticides and mosquito repellents.

## 2. Materials and Methods

### 2.1. Preparation of the Vitamin Solutions and Experimental Animals

A 0.5 g/10 mL aqueous solution of ascorbic acid (AA) (Sigma-Aldrich Chemical Company, St. Louis, MO, USA through Bayouni Trading Co. Ltd., Al-Khobar, Saudi Arabia) was prepared and orally administered through a feeding cannula. α-Tocopherol (E307) (Sigma-Aldrich Chemical Company, St. Louis, MO, USA through Bayouni Trading Co. Ltd., Al-Khobar, Saudi Arabia) in water and 0.2 mL Tween^®^-80 (Sigma-Aldrich Chemical Company, St. Louis, MO, USA through Bayouni Trading Co. Ltd., Al-Khobar, Saudi Arabia) were mixed to produce a total volume of 10 mL. Doses of 100 mg/kg were administered [22]. A total of 40 healthy male albino mice aged 3–4 months and weighing ≈30 g were maintained in 12 h light-dark cycles at a 24 ± 2 °C temperature and relative humidity of 55% with food and water in ample supply. Two weeks of animal acclimatization took place before the start of the experiment was conducted in a closed room of 2.5 m^3^ in size [23].

### 2.2. Toxicant Concentrations, Time-Bound Inhalations, and Exposures

The coil for burning and the liquid and mat-absorbed formulations for vaporization contained 0.05 g, 1.17 g, and 0.04 g of pyrethroids, respectively, which upon exposure for 5 h per day for 21 days amounted to 5.46 g/kg, 21.15 g/kg, and 4.24 g/kg, respectively, for an average 30 g weight of the experimental mice, where all the animals were exposed to a pyrethroid via inhalation through plastic enclosure unit cages (14.5″ × 8.5″ × 5.5″) containing a partition of Perspex sheets, on one side of which the animals were housed, and on the other side, the pyrethroids formulations were placed.

### 2.3. Experimental Design

Male, 3–4-month-old, naïve albino mice (*n* = 40) weighing ≈30 g were obtained from the animal house facility, College of Pharmacy, Qassim University, Saudi Arabia. The animals were maintained in individual polyacrylic cages housing four mice per cage with chow diet (First Milling Company, Qassim, Buraydah, Saudi Arabia) and water ad libitum two weeks before the start of the experiments. The animals were maintained at RT (24 ± 2 °C) and relative humidity of ≈55% with controlled light: dark cycles of 12:12 h. The institutional Research Ethics Committee approved the experimental procedure and the animal care (Approval ID 2019—CP—11) was conducted as per the Guidelines for the Care and Use of Laboratory Animals. Based on the previously published articles, the animals were divided randomly into ten groups (*n* = 4/group) [24,25]. The groups were given 21 days (3 weeks) of exposures and treatments simultaneously. Apart from the group I, all the groups were exposed to commercially available pyrethroid products—e.g., insect/mosquito-repellent pyrethrins containing burning coils; pyrethroid-soaked mats, which released pyrethroids upon heating; and liquid formulations of pyrethroids, which were vaporized for inhalation—for 5 h/day via inhalation. The groups in this study were as follows: group I (control)—no exposure of the animals to toxicants, group II—animal exposure to mat-based vapor, group III—animal exposure to liquid formulation vapor, group IV—burning-coil-based smoke for inhalation by the animals, group V—mat-based vapor for inhalation with 100 mg/kg ascorbic acid (AA) fed *per os* (p.o.), group VI—liquid formulation vapor for inhalation with 100 mg/kg AA fed p.o., group VII—burning-coil-based smoke with 100 mg/kg AA fed p.o., group VIII—mat-based vapor with 100 mg/kg E307 fed p.o., group IX—liquid formulation vapor with 100 mg/kg E307 fed p.o., and group X—burning-coil-based smoke with 100 mg/kg E307 fed p.o.

### 2.4. Sample Collections and Histopathological Examinations

The chloroform-anesthetized animals were sacrificed 24 h after 3 weeks of exposure to different formulations of the pyrethroid toxicants. For the histopathological examinations, animals were laid back, incisions were made in the thoracolumbar region, and body organs were collected and weighed. The left lobe from the liver, apex region of the heart, cortex, and medulla regions from the kidney, lower part of the left lung, and cerebrum and diencephalon regions of the collected brains were washed in normal saline and fixated with 10% aqueous formalin for one consecutive day and night. Samples were treated with automated tissue processors and embedded in paraffin. Tissue sections of 5 μm thickness were sectioned using a microtome. For histological studies, the sections were deparaffinized using xylene and rehydrated in 70% graded ethanol. The sections were mounted on a slide, stained with hematoxylin and eosin, and microscopically examined using a Leica DM500 microscope (Leica Microsystems GmbH, Wetzlar, Germany). The images were scanned using the Leica SCN400 slide scanner (Leica Microsystems GmbH, Wetzlar, Germany).

### 2.5. Data Scoring and Statistical Significance

Thirty fields/sections for each organ were counted. Histopathological data were scored from 0–4, where 0, 1, 2, 3, and 4 represent normal, low, moderate, high, and very high, respectively [26,27]. The Kruskal–Wallis nonparametric test equivalent to the one-way analysis of variance (ANOVA) parametric test for group differences was performed, along with Dunnett’s correction of the significance level for multiple comparisons. Values are represented as mean ± standard error (SE), and 95% confidence intervals (CIs) are represented as lower CI and upper CI. Statistical significance was taken to be *p* < 0.05, and GraphPad Prism version 8.0.2 for Windows (GraphPad, San Diego, CA, USA, www.graphpad.com) was used.

## 3. Results

The pyrethroid-exposed and treated groups’ analysis showed the significant role of anti-oxidants, namely ascorbic acid (AA) and α-tocopherol (EA307), via their ability to ameliorate the toxic effects in the studied groups of experimental animals. The results were comprehensively analyzed using statistical methods, and the values presented in the analysis are expressed as mean ± SE followed by *p*-values obtained from Dunnett’s corrections, as well as 95% confidence intervals (lower CI and upper CI) for each analysis.

### 3.1. Histopathological Findings

The histological findings of the effects of the pyrethrin-formulations exposed, and treated animal groups in relation to the control group of animals are described according to the observations in the specific organs of the animals.

#### 3.1.1. Liver Histopathology

**Figure 2:**
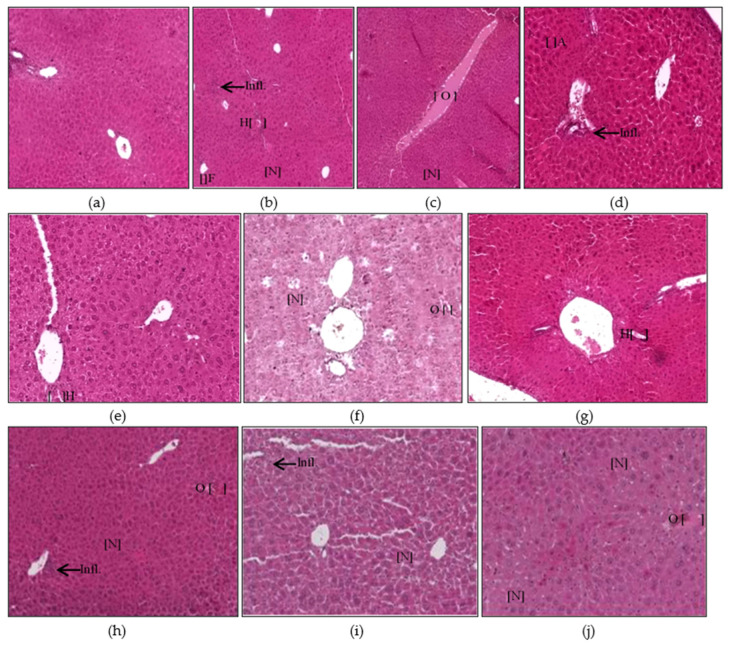
Histopathological photomicrographs (H&E, 400×) of liver tissues showing control (**a**), and the changes after exposure to mosquito repellant (**b**–**d**, Mat-based vapor, Liquid formulation vapor, and Burning-coil-based smoke groups, respectively), and supplementations of pyrethroid-exposed animals with ascorbic acid (**e**–**g**, Mat-based vapor and AA, Liquid formulation vapor and AA and Burning-coil-based smoke and AA groups, respectively), or E307 (**h**–**j**, Mat-based vapor and E307, Liquid formulation vapor and E307, and Burning-coil-based smoke and E307 groups, respectively). The arrows/parentheses in the figures represent connective tissue (CT) infiltration by inflammatory cells (infl), while the other areas marked with abbreviations are necrosis (N), hyperemia (H), fatty changes (F), intracytoplasmic accumulations (A), and occlusion by hyaline material (O).

**Figure 3:**
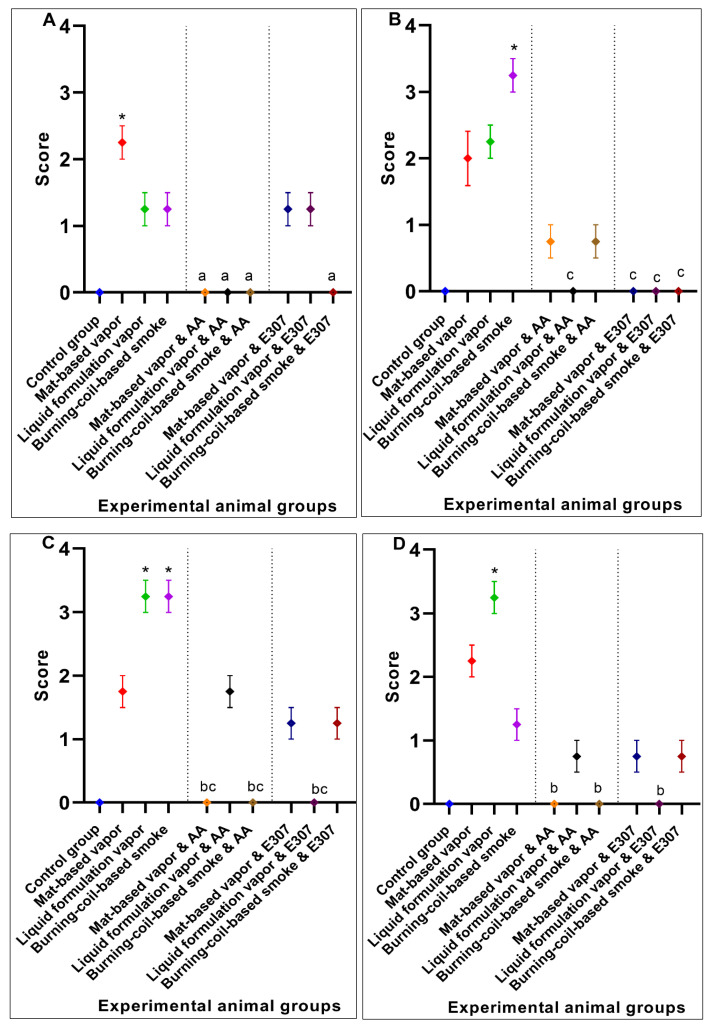
Effects of ascorbic acid (AA) and α-tocopherol (E307) on pyrethroid-induced toxicity in the livers showing inflammation (**A**), hyperemia (**B**), necrosis (**C**), and occlusion by hyaline (**D**) in the animals’ tissue. Values are expressed as mean ± S.E. The Kruskal–Wallis non-parametric test was used for group differences and Dunnett’s corrections of the significance level for multiple comparisons were performed. The significance levels (*p*-values) using Dunnett’s corrections for all the variables were as follows: graph (**A**): *—*p* = 0.02 vs. the control group, while the letter a denotes *p* = 0.02 compared to the animals exposed to mat-based vapor; graph (**B**): *—*p* = 0.02 vs. the control group, while the letter c denotes *p* = 0.02 compared to the animals exposed to burning-coil-based smoke; graph (**C**): *—*p* = 0.01 vs. the control group, while the letters b and c denote *p* = 0.01 compared to the animals exposed to liquid formulation vapor and burning-coil-based smoke, respectively; graph (**D**): *—*p* = 0.01 vs. the control group, while the letter b denotes *p* = 0.01 compared to the animals exposed to liquid formulation vapor.

The control group of animals appeared normal on the histological examinations, and there were no morphological or histopathological alterations in the cardiomyocytes, kidneys, lungs, and brain tissues. Animal exposure to toxicants in mat-based vapor, liquid formulation vapor, and burning-coil-based smoke resulted in severe liver damage, including focal necrosis (N), hyperemia (H), and fatty changes (F) (Figure 2b–d). In comparison to the control group, animal groups exposed to mat-based vapor showed significant inflammation (2.25 ± 0.25, *p* = 0.02, CI: 1.45–3.04), while animals exposed to the liquid formulation vapor showed significant necrosis (1.75 ± 0.25, *p* = 0.01, CI: 0.95–2.54) and occlusion (2.25 ± 0.25, *p* = 0.01, CI: 1.45–3.04). The liver tissue of the animals exposed to coil smoke (group IV) showed intense intracytoplasmic accumulations (A) and hydropic changes characterized by severe necrosis (N), occlusion (O) by hyaline, hyperemia (H), and connective tissue infiltration by inflammatory cells (infl) (Figure 2d) in contrast to the control group. Furthermore, in comparison to the control group (group I), animals exposed to the burning-coil smoke (group IV) showed the highest toxicity with significant hyperemia (3.25 ± 0.25, *p* = 0.02, CI: 1.45–3.04), necrosis (3.25 ± 0.25, *p* = 0.01, CI: 2.45–4.04), and occlusion (1.25 ± 0.25, *p* = 0.01, CI: 0.45–2.04). All the groups treated with AA and E307 showed comparable statuses or insignificant differences for all the measured variables when compared with the control group (Figure 3). The treated animals (groups V–X) showed a decrease in histo-morphological damage compared to the histological findings of animals exposed to mat-based, liquid formulation vapor, and burning-coil-based smoke. The AA-supplemented groups (mat-based vapor and burning-coil-based smoke) showed better results than the E307-supplemented animals for the same set of groups (Figure 3). The animals exposed to mat-based vapor and treated with AA (group V) showed significantly decreased inflammation (0.00 ± 0.00, *p* = 0.02, CI: 0.00 ± 0.00) and occlusion (0.00 ± 0.00, *p* = 0.01, CI: 0.00 ± 0.00) compared to the animals exposed to mat-based vapor, while the animals exposed to liquid formulation vapor and treated with E307 (group IX) showed a significant decrease in necrosis (0.00 ± 0.00, *p* = 0.01, CI: 0.00 ± 0.00) and occlusion of hyaline (0.00 ± 0.00, *p* = 0.01, CI: 0.00 ± 0.00) compared to the animals exposed to the liquid formulation vapor. The animals exposed to the burning-coil-based smoke and treated with E307 (group X) and AA (group VII) showed a significant decrease in hyperemia (0.00 ± 0.00, *p* = 0.02, CI: 0.00 ± 0.00) and necrosis (0.00 ± 0.00, *p* = 0.01, CI: 0.00 ± 0.00) compared to the untreated animals exposed to the burning-coil-based smoke.

#### 3.1.2. Heart Histopathology

**Figure 4:**
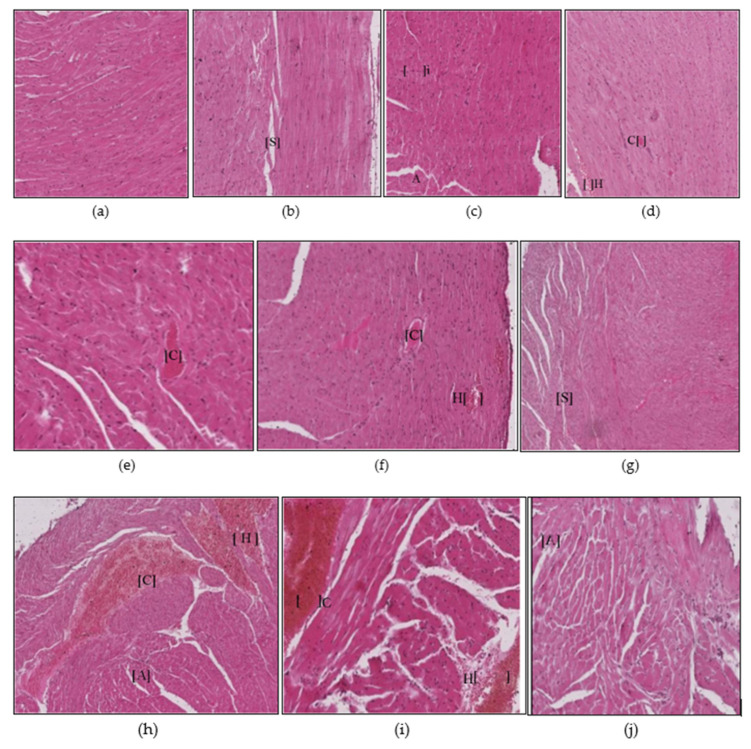
Histopathological photomicrographs (H&E, 400×) of heart tissues showing microscopically observed control (**a**), and the changes after exposure to mosquito repellant (**b**–**d**, Mat-based vapor, Liquid formulation vapor, and Burning-coil-based smoke groups, respectively), and supplementations of pyrethroid-exposed animals with ascorbic acid (**e**–**g**, Mat-based vapor and AA, Liquid formulation vapor and AA, and Burning-coil-based smoke and AA groups, respectively), or E307 (**h**–**j**, Mat-based vapor and E307, Liquid formulation vapor and E307, and Burning-coil-based smoke and E307 groups, respectively). Areas in the figures in parentheses marked with abbreviations represent the separation between cardiac muscle fibers (S), atrophy (A), lymphatic infiltration (i), congestion of blood vessels (C), and hyperemia (H).

**Figure 5:**
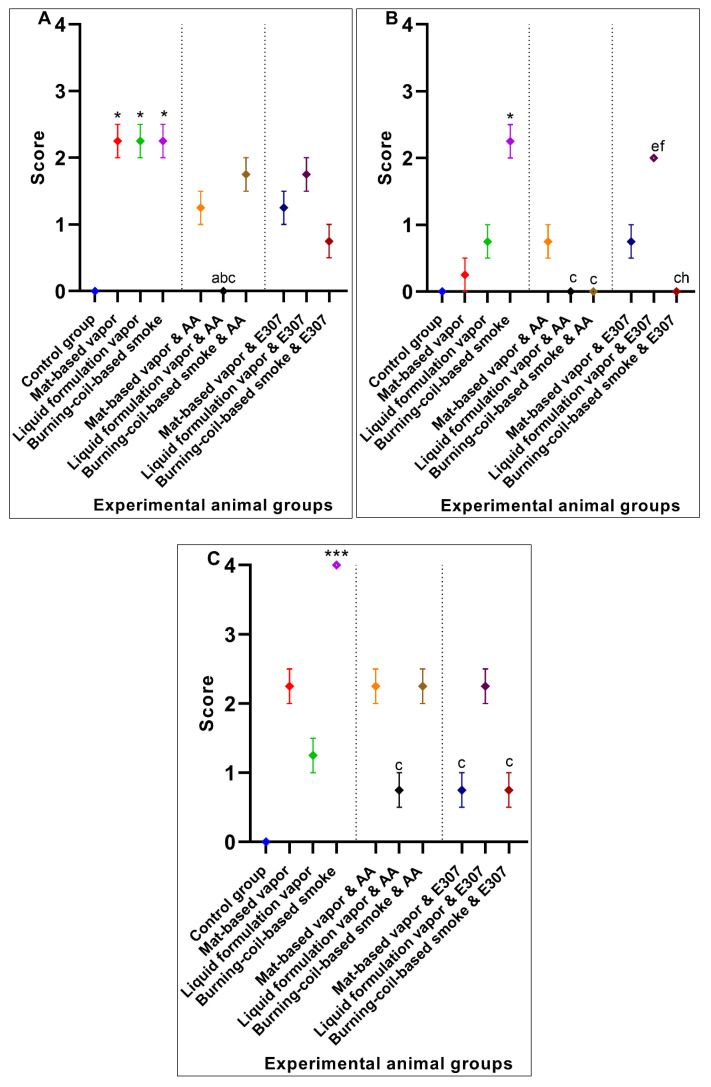
Effects of ascorbic acid (AA) and α-tocopherol (E307) on the pyrethroid-induced toxicity in the animals’ hearts showing atrophy (**A**), congestion of blood vessels (**B**), and hyperemia (**C**). Values are expressed as mean ± S.E. The Kruskal–Wallis non-parametric test was used for group differences and Dunnett’s corrections of the significance level for multiple comparisons were performed. The significance levels (*p*-values) using Dunnett’s corrections for all the variables were as follows: graph (**A**): *—*p* = 0.02 vs. the control group, while the letters denote *p* = 0.03 compared to animals exposed to the mat-based, liquid formulation, and burning-coil-based smoke; graph (**B**): *—*p* = 0.02 vs. the control group, while the letters e and f denote *p* = 0.04 compared to the AA-treated animals exposed to liquid formulation vapor and burning-coil-based smoke. The letters c and h denote *p* = 0.02 compared to the animals treated with E307 and exposed to burning-coil-based smoke and liquid formulation vapor; graph (**C**): ***—*p* < 0.001 vs. the control group, while the letter c denotes *p* = 0.02 compared to the animals exposed to burning-coil-based smoke.

The heart tissues of animals exposed to mat-based and liquid formulation vapor and burning-coil-based smoke showed significant differences in terms of the cardiac muscle fibers (S), atrophy (A), lymphatic infiltration (i), congestion of blood vessels (C), and hyperemia (H) (Figure 4). Among all the formulation types, the animals exposed to coil smoke (group IV) exhibited the most toxicity in the heart tissues with significant atrophy (2.25 ± 0.25, *p* = 0.03, CI: 1.45–3.04), congestion of blood vessels (2.25 ± 0.25, *p* = 0.02, CI: 1.45–3.04), and hyperemia (4.00 ± 0.00, *p* < 0.0001, CI: 4.00–4.00) compared to the unexposed and untreated control group (Figure 5). As such, the animals exposed to burning-coil-based smoke (group VII) treated with AA showed no improvement in atrophy and hyperemia but demonstrated a significant decrease in the congestion of blood vessels (0.00 ± 0.00, *p* = 0.02, CI: 0.00 ± 0.00), while animals exposed to burning-coil-based smoke (group X) treated with E307 showed a significant decrease in the congestion of blood vessels (0.00 ± 0.00, *p* = 0.02, 95% CI: 0.00 ± 0.00) and hyperemia (0.75 ± 0.25, *p* = 0.02, CI: 0.04–1.54) compared to group IV. Furthermore, the animals exposed to mat-based vapor (group II) showed significant atrophy (2.25 ± 0.25, *p* = 0.03, CI: 1.45–3.04) compared to the control group. The animals exposed to liquid formulation vapor (group III) showed significant atrophy (2.25 ± 0.25, *p* = 0.03, CI: 1.45–3.04) in comparison to the control group, but the effect decreased significantly when the animals were treated with AA (group VI, 0.00 ± 0.00, *p* = 0.03, CI: 0.00 ± 0.00) compared to group III. In contrast, the animals exposed to liquid formulation vapor and treated with E307 (group IX) demonstrated no improvement in atrophy or hyperemia. The E307-treated groups’ VIII, IX, and X showed some degree of recovery (Figure 5).

#### 3.1.3. Kidney Histopathology

**Figure 6:**
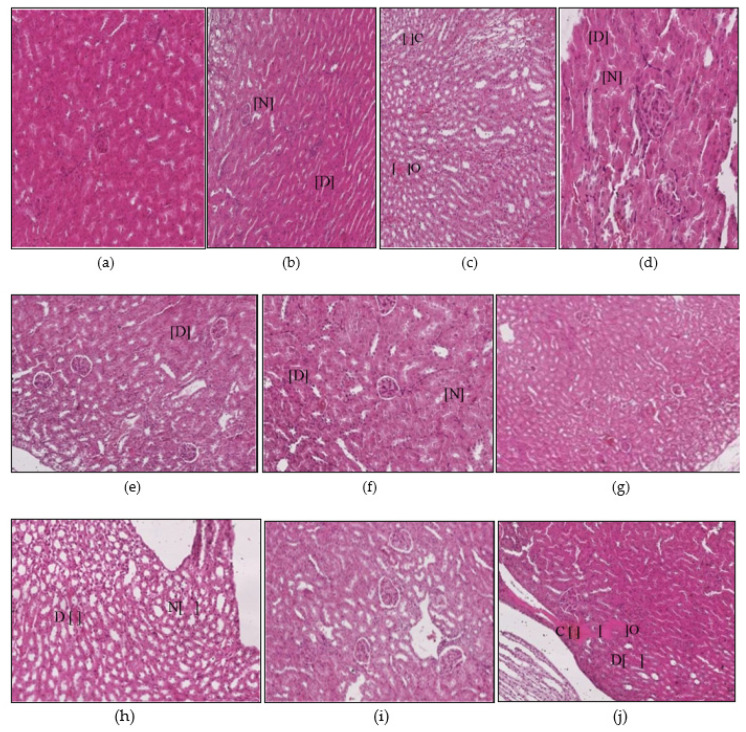
Histopathological photomicrographs (H&E, 400×) of kidney tissues showing microscopically observed control (**a**), and the changes after exposure to mosquito repellant (**b**–**d**, Mat-based vapor, Liquid formulation vapor, and Burning-coil-based smoke groups, respectively), and supplementations of pyrethroid-exposed animals with ascorbic acid (**e**–**g**, Mat-based vapor and AA, Liquid formulation vapor and AA, and Burning-coil-based smoke and AA groups, respectively), or E307 (**h**–**j**, Mat-based vapor and E307, Liquid formulation vapor and E307, and Burning-coil-based smoke and E307 groups, respectively). Areas in the figures marked with abbreviations in parentheses represent the cytoplasmic degeneration of renal tubular cells (D), renal tubules occlusion by hyaline (O), necrotic tubules (N), and congestion and dilation of blood vessels (C).

**Figure 7:**
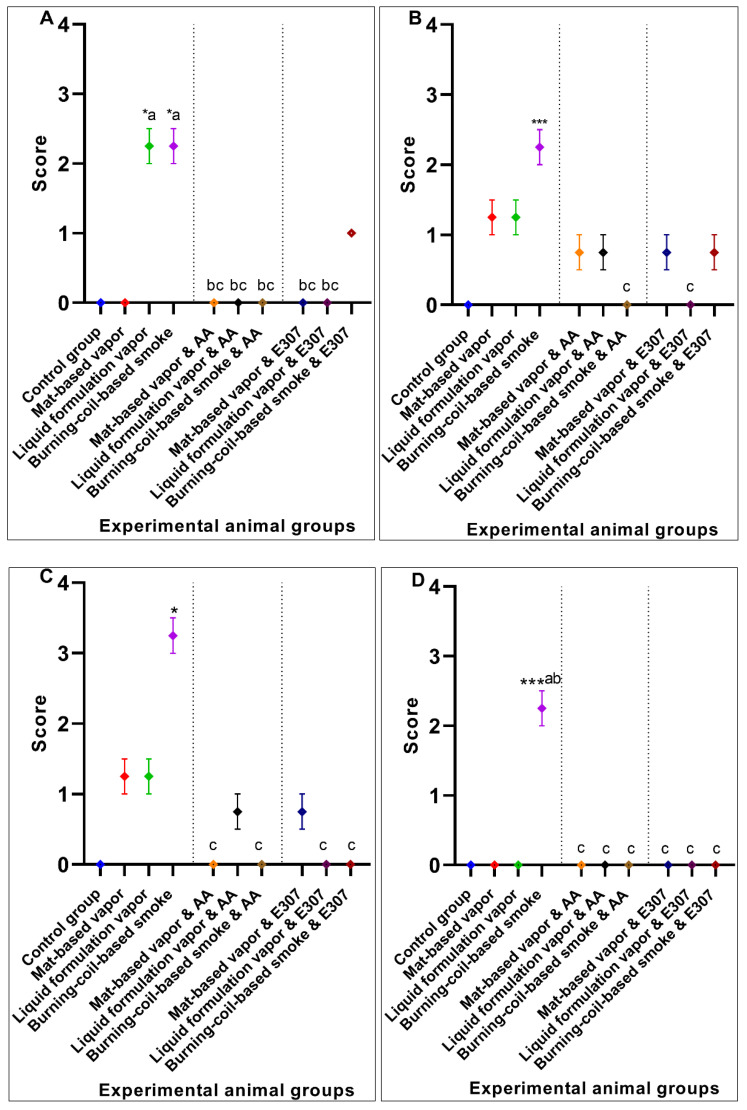

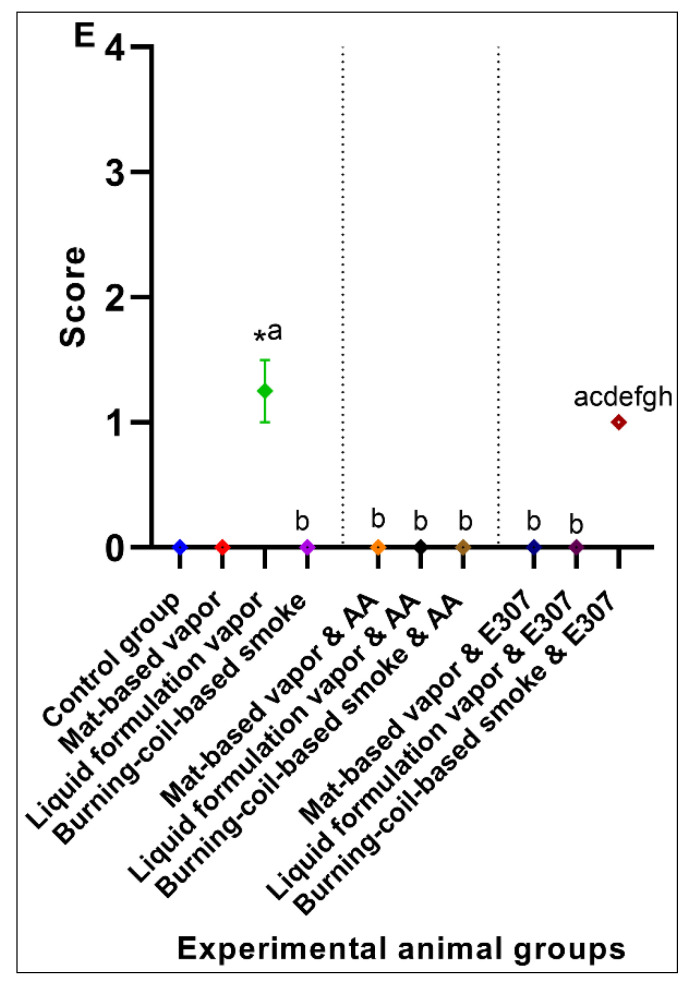
Effects of ascorbic acid (AA) and α-tocopherol (E307) on pyrethroid-induced toxicity in the kidneys of animals showing congestion (**A**), degeneration (**B**), glomerular necrosis (**C**), hyperemia (**D**), and occlusion (**E**). Values are expressed as mean ± S.E. The Kruskal–Wallis non-parametric test was used for group differences, and Dunnett’s corrections of the significance level for multiple comparisons were performed. The significance levels (*p*-values) using Dunnett’s corrections for all the variables were as follows: graph (**A**): *—*p* = 0.04 vs. the control group, while the letter a denotes *p* = 0.04 compared to the animals exposed to mat-based vapor, and the letters b and c denote *p* = 0.04 compared to the animals exposed to liquid formulation vapor and burning-coil-based smoke; graph (**B**): ***—*p* = 0.005 vs. the control group, while the letter c denotes *p* = 0.005 compared to the animals exposed to burning-coil-based coil; graph (**C**): *—*p* = 0.01 vs. the control group, while the letter c denotes *p* = 0.01 compared to the animals exposed to burning-coil-based smoke; graph (**D**): ***—*p* < 0.0001 vs. the control group, while the letters a and b denote *p* < 0.0001 compared to the animals exposed to mat-based and liquid formulation vapor, and the letter c denotes *p* < 0.0001 compared to the animals exposed to burning-coil-based smoke; graph (**E**): *—*p* = 0.01 vs. the control group, while the letter a denotes *p* = 0.01 compared to the animals exposed to the mat-based vapor, the letter b denotes *p* = 0.01 compared to the animals exposed to the liquid formulation vapor, and the letters d–h denote *p* = 0.01 compared with all the treated groups V to IX.

Animals exposed to the toxicants in liquid formulation vapor and burning-coil-based smoke (groups III and IV) revealed modifications regarding the renal cells histology, glomerular tuft necrosis (N), renal tubular cells cytoplasmic degeneration (D), renal tubules occlusion by hyaline (O), necrotic tubules (N), and congestion and dilatation of blood vessels (C) (Figure 6). Among all the toxicity-induced groups, the animals exposed to burning-coil smoke (group IV) expressed severe toxicity in the kidneys with significant congestion (2.25 ± 0.25, *p* = 0.04, CI: 1.45–3.04), cytoplasmic degeneration (2.25 ± 0.25, *p* = 0.005, CI: 1.45–3.04), glomerulus necrosis (3.25 ± 0.25, *p* = 0.01, CI: 2.45–4.04), and hyperemia (2.25 ± 0.25, *p* < 0.0001, CI: 1.45–3.04) compared to the control group (group I), but the effects significantly decreased when the animals were treated with either AA (group VII) or E307 (group X) (Figure 7). Group X treated with E307 demonstrated a significant increase in occlusion. The animals exposed to mat-based vapor (group II) demonstrated the least toxic impacts on the kidneys, and as such, the animals exposed to mat-based vapor and treated with both AA (group V) and E307 (group VIII) showed improvement effects compared to the control group. The animals exposed to the liquid formulation vapor (group III) showed significant congestion (2.25 ± 0.25, *p* = 0.04, CI: 1.45–3.04) and occlusion by hyaline (1.25 ± 0.25, *p* = 0.001, CI: 0.45–2.04) when compared with the control group, but the effects were found to be significantly decreased (0.00 ± 0.00, *p* = 0.04, CI: 0.00 ± 0.00) when animals exposed to liquid formulation vapor were treated with either AA (group VI) or E307 (group IX). Overall, the animals treated with AA and E307 exhibited significant improvements regarding kidney tissue damage.

#### 3.1.4. Lungs Histopathology

**Figure 8:**
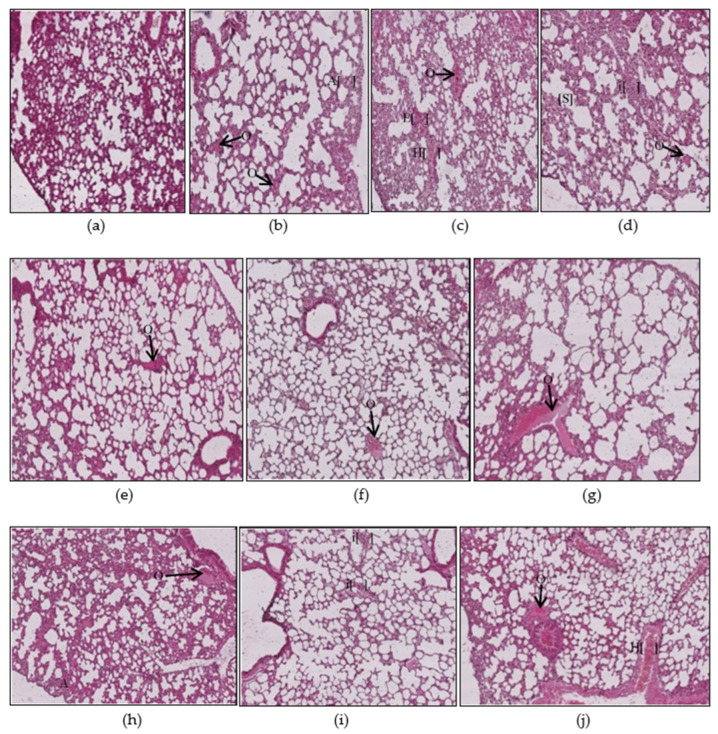
Histopathological photomicrographs (H&E, 400×) of lung tissues showing control (**a**), and the changes after exposure to mosquito repellant (**b**–**d**, Mat-based vapor, Liquid formulation vapor, and Burning-coil-based smoke groups, respectively), and supplementations of pyrethroid-exposed animals with ascorbic acid (**e**–**g**, Mat-based vapor and AA, Liquid formulation vapor and AA, and Burning-coil-based smoke and AA groups, respectively), or E307 (**h**–**j**, Mat-based vapor and E307, Liquid formulation vapor and E307, and Burning-coil-based smoke and E307 groups, respectively). The arrows/parentheses in the figures represent hyaline material occlusion (O), edema (E), enlargement of air spaces (S), interstitial inflammation (i), lymphocyte infiltration aggregates (A), and hyperemia (H).

**Figure 9:**
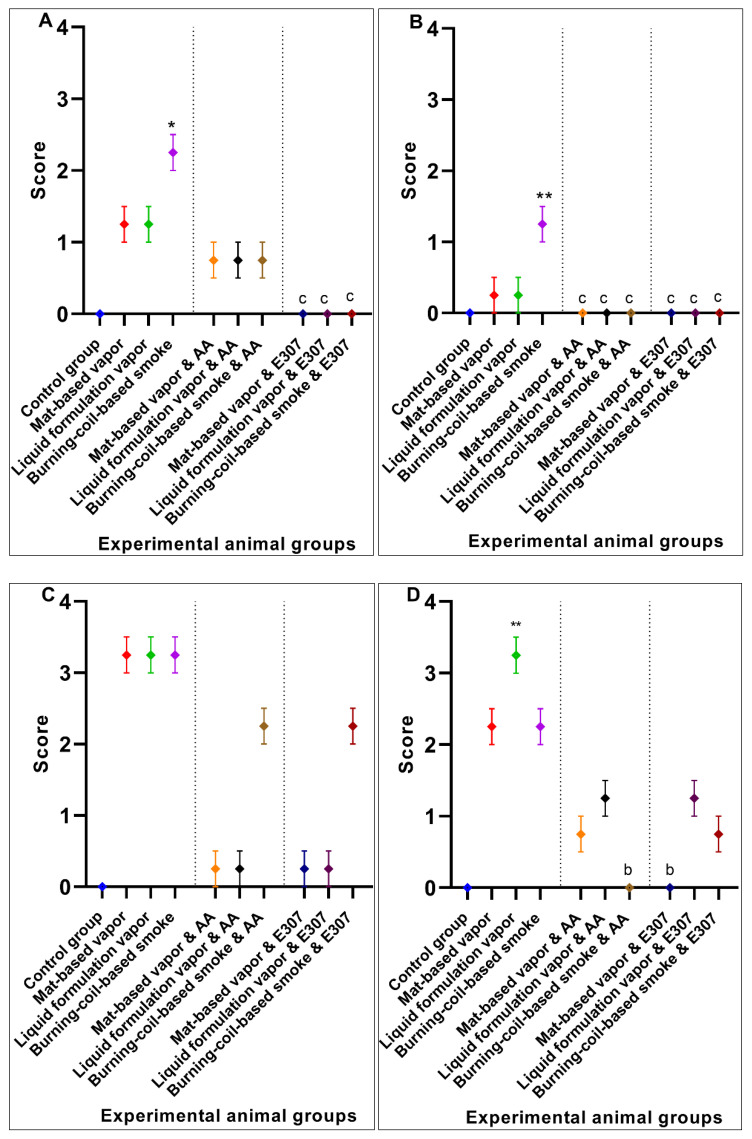

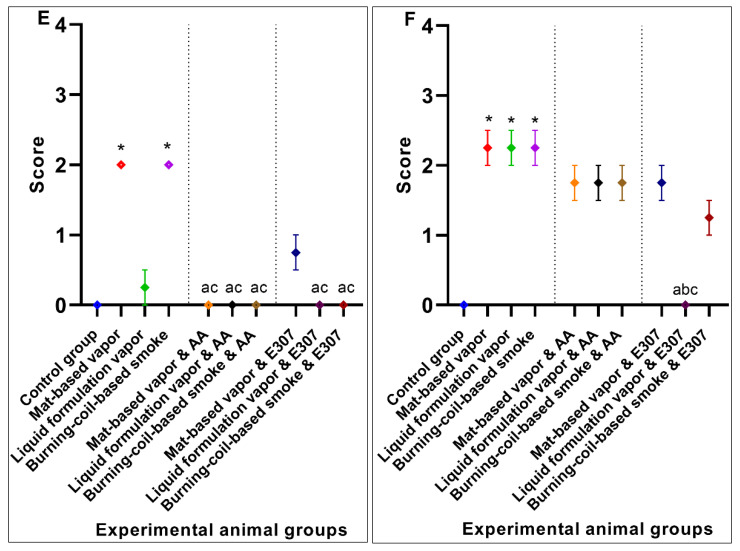
Effects of ascorbic acid (AA) and α-tocopherol (E307) on pyrethroid-induced toxicity in the lungs of the animals showing edema (**A**), enlargement of air space (**B**), hyperemia (**C**), infiltration of CT (connective tissue) by inflammatory cells (**D**), lymphocyte infiltration aggregates (**E**), and occlusion by hyaline (**F**). Values are expressed as mean ± S.E. The Kruskal–Wallis non-parametric test was used for group differences, and Dunnett’s corrections of the significance level for multiple comparisons were performed. The significance levels (*p*-values) using Dunnett’s corrections for all the variables were as follows: graph (**A**): *—*p* = 0.01 vs. the control group, while the letter c denotes *p* = 0.01 compared to the animals exposed to burning-coil-based smoke; graph (**B**): **—*p* = 0.003 vs. the control group, while the letter c denotes *p* = 0.03 compared to the animals exposed to burning-coil-based smoke; graph (**D**): **—*p* = 0.006 vs. the control group, while the letter b denotes *p* = 0.06 compared to the animals exposed to liquid formulation vapor; graph (**E**): *—*p* = 0.04 vs. the control group, while the letters a and c denote *p* = 0.04 compared to the animals exposed to the mat-based and burning-coil-based smoke; graph (**F**): *—*p* = 0.03 vs. the control group, while the letters a–c denote *p* = 0.03 compared to the animals exposed to the mat-based vapor, liquid formulation vapor, and burning-coil-based smoke.

The lung micrographs of the control group demonstrated typical/healthy manifestations of bronchioles, alveoli, and alveolar spaces. The toxicant-exposed animals showed histopathological changes, including hyaline material occlusion (O), edema (E), air-space enlargement (S), interstitial inflammation (infl), lymphocyte infiltration aggregates (A), and hyperemia (H) (Figure 8b–d). All three pyrethroid formulations produced nearly similar effects on the lung tissues. The AA- and E307-treated animals exhibited no apparent improvements in tissue morphology. Some air spaces were still enlarged (Figure 8e–j). The animals exposed to mat-based vapor and treated with E307 (group VIII) revealed recovery from histopathological changes in comparison to other treated groups (Figure 9). Among all the groups, where toxicity was induced, the animals exposed to burning-coil smoke (group IV) expressed the most toxicity with significant edema (2.25 ± 0.25, *p* = 0.01, CI: 1.45–3.04), enlargement of air space (1.25 ± 0.25, *p* = 0.03, CI: 0.45–2.04), lymphocyte infiltration aggregates (2.00 ± 0.00, *p* = 0.04, CI: 2.00–2.00), and occlusion by hyaline (2.25 ± 0.25, *p* = 0.03, CI: 1.45–3.04) compared to the control group, but the enlargement of the air space decreased significantly (0.00 ± 0.00, *p* = 0.03, I: 0.00 ± 0.00) when treated with AA (group VII), while the edema decreased significantly (0.00 ± 0.00, *p* = 0.01, CI: 0.00 ± 0.00) when treated with E307 (group X) (Figure 9). The animals exposed to mat-based vapor (group II) demonstrated a significant increase in lymphocyte infiltration aggregates (2.00 ± 0.00, *p* = 0.04, CI: 2.00–2.00) and occlusion by hyaline (2.25 ± 0.25, *p* = 0.03, CI: 1.45–3.04), while the animals exposed to the liquid formulation vapor showed an increase in the infiltration of CT (connective tissue) by inflammatory cells (3.25 ± 0.25, *p* = 0.006, CI: 2.45–4.04) and occlusion by hyaline (2.25 ± 0.25, *p* = 0.03, CI: 1.45–3.04). All the untreated toxicity-induced animal groups showed increased hyperemia compared to the control group, while the animals exposed to mat-based and liquid formulation vapor treated with either AA or E307 showed complete recovery (Figure 9C).

#### 3.1.5. Brain Histopathology

**Figure 10:**
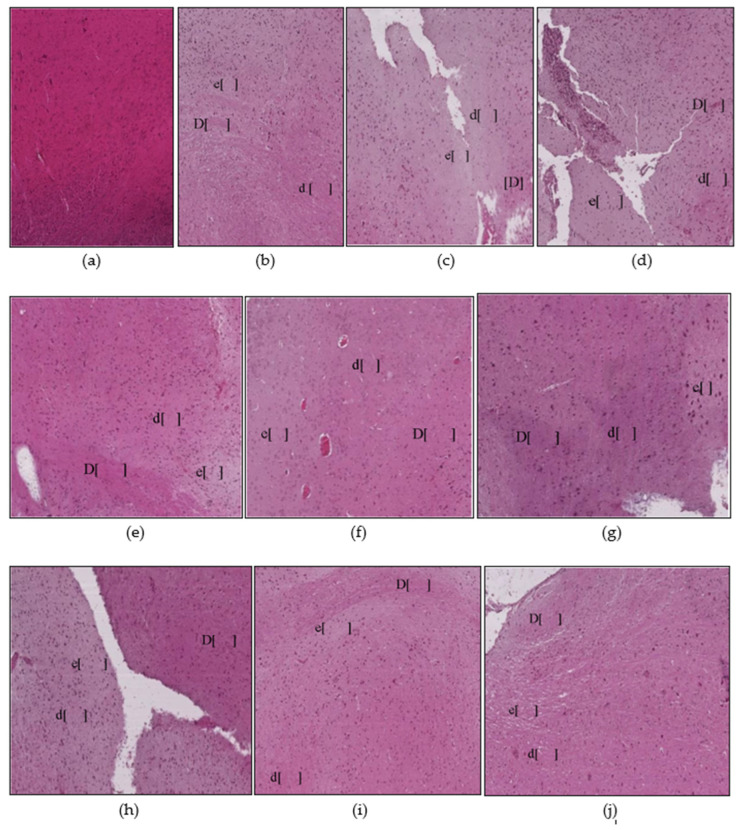
Histopathological photomicrographs (H&E, 400×) of brain tissues showing control (**a**), and the changes after exposure to mosquito repellant (**b**–**d**, Mat-based vapor, Liquid formulation vapor, and Burning-coil-based smoke groups, respectively), and supplementations of pyrethroid-exposed animals with ascorbic acid (**e**–**g**, Mat-based vapor and AA, Liquid formulation vapor and AA, and Burning-coil-based smoke and AA groups, respectively), or E307 (**h**–**j**, Mat-based vapor and E307, Liquid formulation vapor and E307, and Burning-coil-based smoke and E307 groups, respectively). Abbreviations in the figures with parentheses represent nervous tissue degeneration (D), decreased number of neurons (d), and decreased uptake of eosin (e).

**Figure 11:**
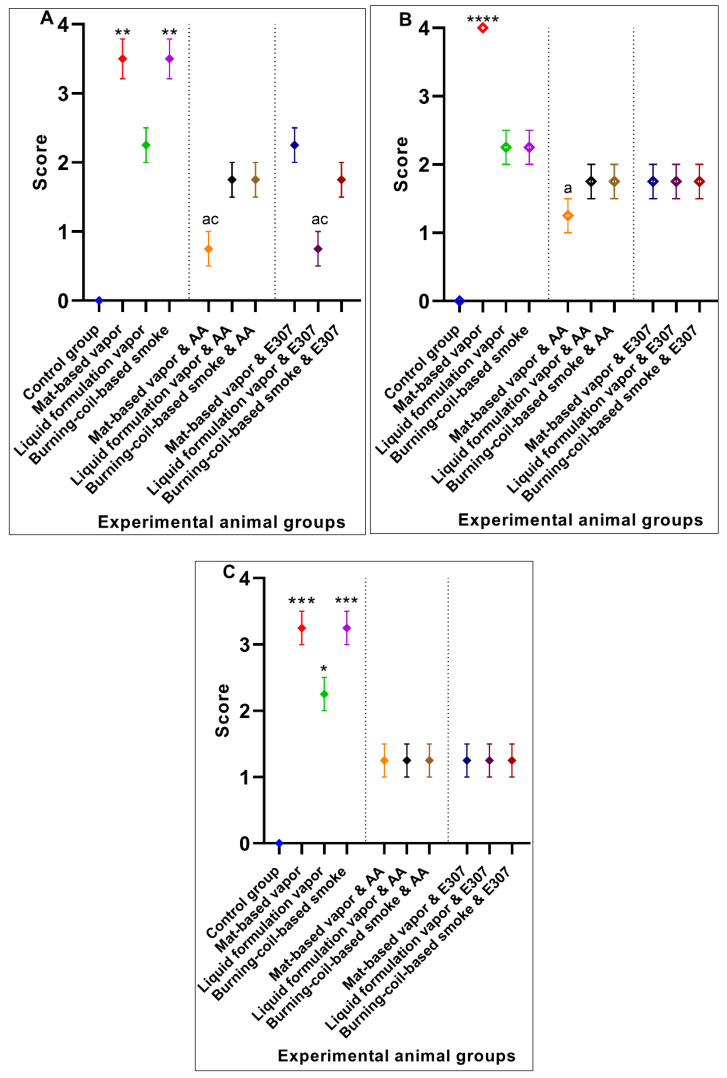
Effects of ascorbic acid (AA) and α-tocopherol (E307) on pyrethroid-induced toxicity in the brain of the animals with the degeneration of nervous tissue (**A**), lack of eosin uptake (**B**), and decrease in the number of neurons (**C**). Values are expressed as mean ± S.E. The Kruskal–Wallis non-parametric test was used for group differences and Dunnett’s corrections of the significance level for multiple comparisons were performed. The significance levels (*p*-values) using a Dunnett’s corrections for all the variables were as follows: graph (**A**): **—*p* < 0.002 vs. the control group, while the letters a and c denote *p* = 0.03 compared to the animals exposed to mat-based vapor and burning-coil-based smoke; graph (**B**): ****—*p* < 0.0001 vs. the control group, while the letter a denotes *p* = 0.02 compared to animals exposed to mat-based vapor; graph (**C**): *—*p* = 0.03 and ***—*p* < 0.001 vs. the control group.

The control group (Figure 10a) animals showed regular and compacted arrangements of brain tissues, whereas the animals exposed to toxicants in mat-based vapor displayed significant nervous tissue degeneration (D), a decrease in the number of neurons (d), and decreased uptake of eosin (e) compared to the control group. Among all the toxicity-induced groups (Figure 10b–j), the animals exposed to mat-based vapor (group II) expressed the most toxicity in the brain with significant degeneration of nervous tissue (3.50 ± 0.28, *p* = 0.002, CI: 2.58–4.41), lack of eosin uptake (4.00 ± 0.00, *p* < 0.0001, CI: 4.00–4.00), and decreased number of neurons (3.25 ± 0.25, *p* < 0.001, CI: 2.45–4.04) compared to the control group (group I), but the degeneration of nervous tissue (0.75 ± 0.25, *p* = 0.03, CI: 1.00–1.00) and lack of eosin uptake effects decreased significantly (1.25 ± 0.25, *p* = 0.02, CI: 0.45–2.04) when animals were treated with AA (group V) (Figure 11). Similarly, the animals exposed to liquid formulation vapor (group III) showed a significant decrease in the number of neurons (2.25 ± 0.25, *p* < 0.001, CI: 1.45–3.04), while animals exposed to burning-coil-based smoke (group IV) also showed a significant decrease in the number of neurons (3.25 ± 0.25, *p* < 0.0001, CI: 2.45–4.04). The animals exposed to liquid formulation vapor and treated with AA and E307 (groups VI and IX) and the animals exposed to burning-coil-based smoke (group VI and X) showed no improvements in the uptake of eosin.

### 3.2. Significant Effects of AA and E307 on the Experimental Animals’ Tissues

In the liver tissues, the animals exposed to mat-based vapor and treated with AA and animals exposed to burning-coil-based smoke (groups V and VII) showed no necrosis, inflammation, or occlusion, and were observed to have low hyperemia compared to the E307-treated animals (Figure 2e,g). In the heart tissues, both AA- and E307-treated animals showed some degree of recovery. However, among all the treated groups, the animals exposed to the liquid formulation vapor and treated with AA (group VI) showed the greatest recovery in their heart tissues (Figure 4f). In the kidneys, the animals exposed to mat-based vapor and burning-coil-based smoke that were treated with AA, and the animals exposed to liquid formulation vapor and treated with E307 (group IX) showed the most significant recoveries of their congestion, degeneration of renal tubules, and glomeruli (Figure 6i). The animals exposed to liquid formulation vapor and treated with E307 (group VIII), followed by animals exposed to mat-based vapor (group VIII), showed the greatest recovery in their lung tissues (Figure 8h). However, the animals exposed to mat-based vapor and treated with AA (group IV) showed a much-improved morphology with improved uptake of eosin and fewer brain tissue degenerations. A representation of the comparative organ damage is presented in Table 1. Among all the toxicity-induced groups, the animals exposed to mat-based vapor demonstrated the greatest beneficial improvements in the liver, kidney, and brain, while treatments did not induce similar beneficial impacts compared to other pyrethroid formulation exposures in the lungs and heart tissues.

## 4. Discussion

### 4.1. Pyrethroid Toxicity, Treatment, and Role of Ascorbic Acid and α-Tocopherol: Histopathological Examinations

The histopathological examinations revealed the effects on the liver, heart, kidneys, lungs, and brain of the mice (Figure 2, Figure 3, Figure 4, Figure 5, Figure 6, Figure 7, Figure 8, Figure 9, Figure 10 and Figure 11). The pyrethroids exposure demonstrated glomerular tuft necrosis, degeneration of renal tubular cells, renal tubules occlusion by hyaline, necrotic tubules, and congestion of blood vessels (Figure 7) [28,29]. The congested blood vessels and degenerative changes led to the observed renal parenchymal tissue’s weakness. The renal tissue fibrosis, multifocal congestion, and cystic dilation in kidney tissue of the animals exposed to pyrethroids were observed. The animals exposed to mat-based vapor and treated with AA (group IV) showed glomerular tuft necrosis degeneration of their renal tubular cells. An earlier study [30] also confirmed similar observations that lead to conclude the nephrotoxicity of the pyrethroids.

The lung tissues of the experimental mice treated with pyrethroids showed infiltration of their connective tissues by inflammatory cells, hyaline material occlusion, and hyperemia, as was also observed earlier [28]. The edema in the current study was possible due to the inflammation generated from the irritation of the lungs’ tissues. Another synthetic pyrethroids product, cypermethrin, produces morphological alterations in the brain and heart tissues, leading to hypoxia, neuronal degeneration, and necrosis with elevated glial cells in the brain tissues. It also causes necrosis of heart tissue and generates metabolite toxicities and myocardial defects. An earlier study [31] also confirmed neuronal damage. Atrophy and hyperemia were also observed in heart tissue. The histological examination of kidneys, livers, and lungs revealed severe organ damages, which showed widespread hepatocyte degenerations, congested blood vessels, fatty changes, infiltration of inflammatory cells, and fibrosis, as was also confirmed by previous observations [32]. The intense intracytoplasmic accumulations and hydropic changes were characterized by severe necrosis, occlusion by hyaline material, hyperemia, and connective tissue (CT) infiltration by inflammatory cells. Cypermethrin generates toxic effects in hepatocytes in two different ways: (1) it produces reactive oxygen species (ROS) that induce oxidative stress and (2) it builds-up in cell membranes because of its hydrophobic character, thereby disturbing the cell membranes. The resultant oxidative stress initiates and advances liver injury, whereupon AA and E307 seemingly reduce the histopathological changes generated by the pyrethroids due to their antioxidant properties, where these changes were observed in the present study.

This study investigated the ameliorating effects of both AA and E307, as assessed through histopathological observations of the affected organs. Earlier workers have reported the protective effects of AA on certain renal parameters caused by pyrethrins, as well as cypermethrin-induced nephrotoxicity [32]. AA’s role in ameliorating different toxicities is known. However, its protection against lead toxicity is inconclusive [33]. In another report, the preventive effects of AA in chromates-based toxicity is well documented [34], including for certain cancers [35]. The contribution of AA in the prevention and cure of various ailments is available [36]. The significant cardioprotective effects of AA on doxorubicin-induced myocardial toxicity in experimental animals are also known [37]. The protective effects of E307 were observed in patients treated with radiological interventions for gynecologic tumors [38]. E307, in combination with pentoxifylline, was effective at preventing and reducing the toxicity caused by radiation [39]. The E307-induced toxicity reduction was also observed in gynecologic tumors for patients with radiotherapy [34]. Chemically induced toxicity was also controlled through the use of vitamin E (α-tocopherol, equivalent E307) [40]. An expert-evaluated report on α-tocopherol and its various derivatives as cosmetological ingredients also vouched for the product’s safety for human use [41].

In our study, the AA-treated groups showed improved morphology of the affected brain tissues, with significantly improved numbers of neurons with the least tissue degeneration. The AA was also shown to decrease histomorphological damages in the brain tissues in comparison to liver damage, also observed earlier [42,43,44,45,46,47].

### 4.2. Molecular Mechanism: A Plausible Biomechanics Outline Proposed for Toxicity Generation, and Its Amelioration

AA (ascorbic acid), a water-soluble antioxidant, plays a vital role in liver metabolism, collagen formation hemopoiesis, and other metabolic functions. It is important for protection from insecticidal- and pesticide-based toxicity, in the liver as an antioxidant, and to evade the consequences of free radicals. It scavenges hydroxyl and superoxide radicals and is principally active in plasma and cytosine [48,49,50]. In our study, AA and E307 (α-tocopherol) administration to the pyrethroid-exposed animals resulted in a decrease in the histomorphological damage compared to non-AA/non-α-tocopherol-supplemented animals (groups II, III, and IV).

Plausible mechanistic steps in toxicity remediation involving AA and α-tocopherol in various stages of malfunctioning; tissue damage; inflammation; hypermeria; necrosis; eventual cell degeneration; chemical-based adverse and allergic reactions, including induction of immune enhancement; speeding up of the wound-healing; stopping of free-radical damage are also known [35,36]. The AA is known to work on the heart through anti-oxidant enzymes and reduces cell damage severity [37]. Continuous feeding with AA maintains the effectiveness of the inherent anti-oxidant system through reactive oxygen species scavenging in the affected area. The α-tocopherol is known to reduce inflammation and protect the hepatocytes from adriamycin-induced damage in cancer patients [37,38,39]. In the present study, all the AA-fed groups were better controlled in their toxic damage caused by different formulations of pyrethroids, which is conclusively evident from the protective roles of AA.

The neuroinflammation and brain toxicity affecting the neurons (and the glial cells) leading to degeneration is linked with the reactive oxygen species (ROS) presence, which in this case of exposures is uncontrolled [48,49,50]. It is known that free radicals and ROS lead to protein, nucleic acid, and lipid oxidations [51]. A weakening of the endogenous antioxidant defense system (EAS) is presumably responsible for the toxicity and tissue damages, wherein the presence of AA and E307 plays a part in ameliorating toxic effects. The copper-zinc and manganese superoxide dismutase (CuZnSOD, MnSOD) [52], catalase, and glutathione peroxidases (GSHPx) [53,54] are some of the biomarkers indicating the effectiveness of an anti-oxidant system. On another front, the free radical generation is the starting point of the chemical induction, which in this case, was pyrethroids, toxicity, and organ malfunctions and damage [55,56]. The free radical is generated by the spin-impairment of electron pairs, which is achieved by external and internal factors, one of them being the chemical-induced stress [57]. The produced entities are oxygen radical (O^•^), superoxide (O_2_^•−^), hydroxyl (OH^•^), and nitric oxide (NO^•^) free radicals [58]. Furthermore, the outside factors trigger the xanthine oxidase (XO) and NADPH oxidase-2 (NOX-2) systems. The heightened levels of xenobiotic chemicals, either as vapor or as dissolved-form entities tend to lower the cellular oxygen concentrations, leading to faster ATP metabolism and the deposition of hypoxanthine. Nonetheless, the oxygen reperfusion triggers XO, thereby reducing the hypoxanthine to xanthine and producing the superoxide and H_2_O_2_ [59,60] entities. The NADPH oxidase (NOX^−2^), a complex of cytochrome b558 (p22^PHOX^, gp91^PHOX^), cytosolic proteins (p40^PHOX^, p47^PHOX^, and p67^PHOX^), and Rac G-protein [61] produce superoxide free-radicals via the phosphorylation of cytosolic proteins and Rac activations. Superoxide-induced arginine metabolism via the nitric oxide synthases (NOS) helps to produce ONOO^−^, which, together with the hydrogen peroxide, are transformed to free radicals responsible for the neurodegenerations. The oxidative modifications and damage to proteins lead to a reduction in their functional competencies and stability [62,63], thereby generating proteopathy [64], malfunctioning, and organ damage. The mitochondrial damage and dysfunctions, together with disturbances in the chemical setup, are responsible factors involved in the toxicity [65,66,67]. It is known that antioxidants help to control the adverse effects of free radicals, ROS, and the levels of glial activation [68].

**Figure 12:**
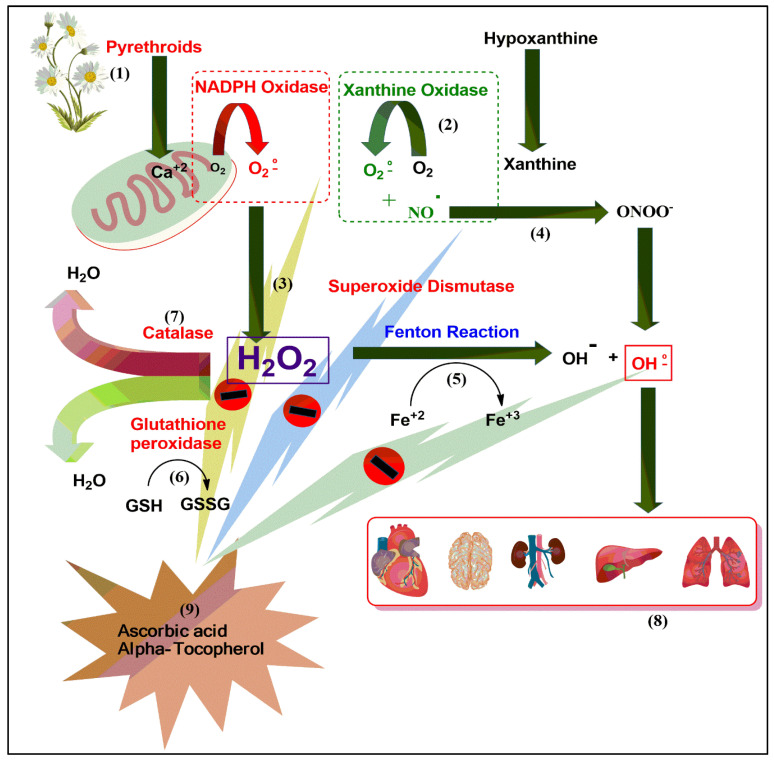
The oxidative stress cascade mechanism illustrating the toxicity generation sequence and the AA and E307 inhibitory actions on the free radicals.

E307 interrupts the harmful oxidations and cleanses the free radicals [69]. Both AA and E307 minimize the free-radical counts by scavenging them through their anti-oxidant actions and thus prevent liver damage [27]. The ameliorating effects are primarily provided by antioxidant compounds that scavenge the generated radicals, along with quenching the reactive oxygen and nitrogen species, nitric oxide radical, and nitric oxide super radical. A closer look revealed the strong anti-oxidant characteristics of AA in comparison to E307 (α-tocopherol) (Table 1), as was also shown in the histological examination observations, which together confirmed the highly positive toxicity amelioration of the AA-fed groups in this pilot study. A brief mechanistic outline is represented in Figure 12, proposing the major steps in the toxicity generation.

In a nutshell, the generation of ROS and the role of the Fenton reaction and enzymatic activities, as well as the biochemical catalysis and quenching of the radicals by the anti-oxidants AA and E307 are depicted to show the damage and amelioration processes.

## 5. Conclusions

The histopathological examinations revealed that the toxicity amelioration in the liver, the occlusion by hyaline, necrosis, and inflammation in the AA-fed animals exposed to mat-based vapor and burning-coil-based smoke were comparable to the animals of the control group. The AA-supplemented groups also showed significantly better recovery results than the E307 (α-tocopherol)-fed groups. In the heart, the congestion of blood vessels for both the AA- and E307-fed animals exposed to burning-coil-based smoke was also comparable to the animals of the control group in terms of damage recovery.

In the kidneys, all the observed functional variables for the kidney recovery/organ damage ameliorations were comparable to the control group for both the AA-fed animals exposed to burning-coil-based smoke and the E307-fed animals exposed to liquid formulation vapor. Furthermore, the recoveries of both the AA- and E307-fed animals exposed to mat-based vapor and animals exposed to liquid formulation vapor were also comparable to the animals of the control group in terms of the observed congestions, hyperemia, and occlusions repairs.

Additionally, in the lungs, all AA- and E307-treated groups showed the greatest damage amelioration in terms of the enlargement of air-space recovery and histopathological examination comparable to the conditions of the control group tissues. Similarly, the hyperemia score was comparable for both the AA- and E307-fed animals exposed to mat-based vapor and animals exposed to liquid formulation vapor. Furthermore, the edema and lymphocyte infiltration aggregates for all the treated groups of E307- and AA-fed animals, respectively, were comparable to the control group in terms of the observed recovery, as observed in the microscopic examinations. Again, all the observed variables, except lymphocyte infiltration aggregates, displayed the same recovery in the exposed and treated animals as the control group of animals. This was also true for the animals exposed to liquid formulation vapor, and the supplements-fed groups of damage-recovered animals. In the brain, none of the observed variables for both the AA- and E307-fed and treated animal groups were comparable to the animals of the control group.

**Figure 13:**
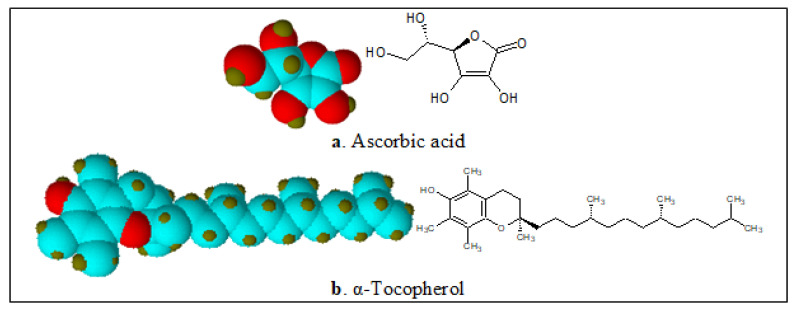
Structures and 3D models of ascorbic acid (AA) and E307 (α-tocopherol) molecules.

The adverse effects upon chronic exposure and inhalations of the pyrethroid formulations led to severe damage in multiple organs, i.e., liver, kidneys, lungs, heart, and brain, where this damage was ameliorated by AA and E307 (Figure 13) supplementations to the animals to varying degrees, and the toxicities were overcome in the majority of treated animal groups.

**Figure 14:**
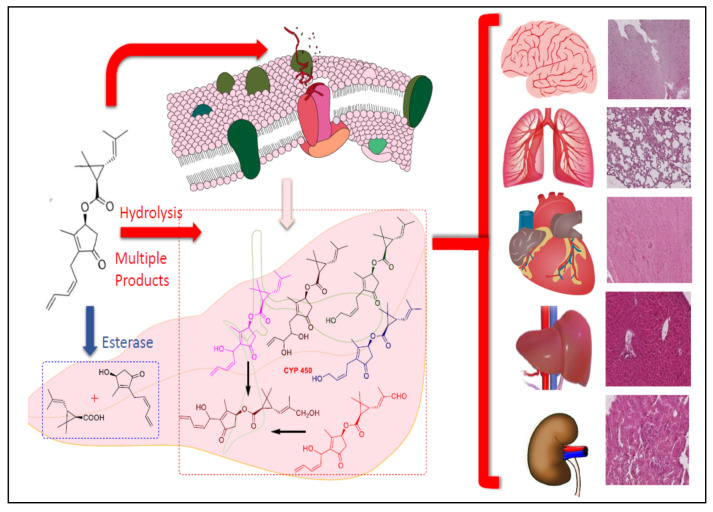
A representation of the metabolism of pyrethrins and organ damage.

A representational depiction of the metabolism of pyrethrins and organ damage effects is presented in Figure 14.

The study has the potential for complete amelioration of the toxicity through, probably, an increased dose and duration of the treatment with vitamins feeds. The findings also suggested a plausible prophylactic use of the anti-oxidants, especially ascorbic acid and α-tocopherol, for preventing the pyrethroid-induced damage in humans.

## Figures and Tables

**Table 1:** Predominant effects of AA and E307 on the tissues of experimental animals.

Organ	The Group with Significant Recovery	Significant Compound
Liver	AA-treated mat-based-vapor exposure	AA (Ascorbic Acid)
	AA-treated burning-coil-based smoke exposure	AA
Heart	AA-treated liquid formulation vapor exposure	AA
Kidneys	AA-treated mat-based vapor exposure	AA
	AA-treated burning-coil-based smoke exposure	AA
	E307-treated liquid formulation vapor exposure	E307 (α-Tocopherol)
Lungs	E307-treated liquid formulation vapor exposure	E307
Brain	AA-treated mat-based vapor exposure	AA
